# Increased Seroreactivity to Glioma-Expressed Antigen 2 in Brain Tumor Patients under Radiation

**DOI:** 10.1371/journal.pone.0002164

**Published:** 2008-05-14

**Authors:** Sabrina M. Heisel, Ralf Ketter, Andreas Keller, Veronika Klein, Christian P. Pallasch, Hans-Peter Lenhof, Eckart Meese

**Affiliations:** 1 Department of Human Genetics, Saarland University Medical School, Homburg/Saar, Germany; 2 Neurosurgical Clinic, Saarland University Medical School, Homburg/Saar, Germany; 3 Center for Bioinformatics, Saarland University, Saarbrücken, Germany; 4 Internal Medicine Clinic I, University of Cologne Medical School, Cologne, Germany; Centre de Recherche Public-Santé, Luxembourg

## Abstract

**Background:**

Surgery and radiation are the mainstays of therapy for human gliomas that are the most common primary brain tumors. Most recently, cell culture and animal studies provided the first convincing evidence that radiation not only eliminates tumor cells, but also modulates the immune response and likely improves anti-tumor immunotherapy.

**Methology/Pricipal Findings:**

We present an *in vivo* study that analyzes the effects of radiation on the immune response in tumor patients. As readout system, we utilized the reactivity of glioma patients' sera against antigen GLEA2 as the most frequent antigen immunogenic in glioblastoma patients. We established an ELISA assay to analyze reactivity of 24 glioblastoma patients over a period of several months. As control we used 30 sera from healthy donors as well as 30 sera from lung cancer patients. We compared the course of GLEA2 seroreactivity at different times prior, during and after radiation. The GLEA2 seroreactivity was increased by the time of surgery, decreased after surgery, increased again under radiation, and slightly decreased after radiation.

**Conclusions/Significance:**

Our results provide *in vivo* evidence for an increased antibody response against tumor antigens under radiation. Antigens that become immunogenic with an increased antibody response as result of radiation can serve as ideal targets for immunotherapy of human tumors.

## Introduction

Malignant gliomas that are derived from the glial lineage represent a major class of tumors of the central nervous system (CNS) with glioblastoma multiforme (GBM) as the most common malignancy of the CNS [1]. Treatment is almost never curative even for patients with low-grade gliomas. Two-year survival for patients with glioblastoma is less than 30% [1]. The rate of overall survival and disease-free survival did not change appreciably over three decades.

Surgery, radiation and recently temozolomide chemotherapy have been the basis of therapy [2]–[4]. Other approaches such as immunotherapy have yet to find their way into clinical praxis. Known consequences of ionizing radiation include induction of double strand DNA breaks, DNA and protein modification by radical formation [5], [6]. Now, it has been shown that radiation can also modulate the peptide repertoire and enhance the MHC class I expression [7]. These recent data indicate possibilities that radiation cannot only be used to eliminate tumor cells, but also to modify the immune response. As a result of radiation, the tumor cells may increasingly present specific antigens. These antigens can subsequently be targeted by immunotherapy.

There are only few immunogenic antigens that have been reported for gliomas [8]–[12]. For our study we analysed glioma-expressed antigen 2 (GLEA2) that shows the most frequent antibody response in glioma patients [13]. We compared GLEA2 seroreactivity by ELISA prior and after radiotherapy of glioblastoma patients.

## Materials and Methods

### Patients

Patients eligible for this study were 18 to 75 years of age, with a histological proven glioblastoma multiforme and a Karnofsky Performance Score of 70 or better. Patients with renal, hepatic or bone marrow impairment, HIV infection, prior chemotherapy or stereotactic biopsy were excluded.

In total, 24 cases of newly diagnosed glioblastomas operated during the period of March 2004 through April 2005 were studied. All patients (14 males and 10 females) underwent radical tumor resection. The median patient age was 56.8 years with a range from 36.9 to 72.5 years. In all cases surgery was followed by radiotherapy that consisted of fractionated focal irradiation at a dose of 1.8–2 gray (Gy) per fraction given once daily five days per week over a period of 6 weeks, for a total dose of 60 Gy. Radiotherapy was delivered to the gross tumor volume with a 2 cm margin volume for the clinical target volume based on a preoperative magnetic resonance image (MRI). In 17 cases patients additionally underwent chemotherapy treatment consisting of temozolomide (marketed as Temodal® in Europe and Canada and Temodar® in the United States; Schering-Plough). In nine cases chemotherapy was applied concomitant to radiotherapy at a dose of 75 mg/m^2^/d, given 7 days per week from the first day of radiotherapy until the last day of radiotherapy, but for no longer than 49 days. After a 4-week break, patients received up to six cycles of adjuvant temozolomide every 28 days according to the standard 5-day schedule [4]. In the remaining cases the radiotherapy regime was followed by an adjuvant chemotherapy that was administered at a dose of 150 mg/m^2^/d on day 1–5 in the first cycle. The following cycles were done at a dosage of 200 mg/m^2^/day. Treatment cycles were repeated every 28 days.

The baseline examination included computer tomography (CT) or magnetic resonance imaging (MRI), full blood counts and blood chemistry tests, and a physical examination. All patients were to be seen every 4 weeks and blood samples were collected. Due to neurological deficits, some of the patients were not examined in our outpatient department, but in their own home.

We obtained ethical approval from local ethics committee for “Development of minimal invasive glioma diagnostics” (ethical approval No. 67106) involving both, Department of Human Genetics, Saarland University and Neurosurgical Clinic, Saarland University Medical School. All patients gave written informed consent for the use of their blood samples for analysis.

### Plasmids and cell lines

For baculovirus expression system, we used pBlueBacHis 2a (Invitrogen, Rockville, MD) to express 6xHis-tagged GLEA2 N-terminus. Spodoptera frugiperda cell line Sf158 was cultured in TC100 medium (Invitrogen) supplemented with 10% FBS and 1% penicillin-streptomycin (Invitrogen) at 27°C in non-humidified environment.

### Protein expression and purification

Infected SF158 cells were incubated for 46 h at 27°C, detached with a cell scraper and centrifuged for 15 min at 3.000 rpm. The cell pellet was resuspended in lysis buffer (50 mM NaH_2_PO_4_ (pH 8.0), 300 mM NaCl, 5 mM imidazole, 1% NP40, protease inhibitor cocktail (Roche, Mannheim, Germany)) and incubated on ice for 20 min. The lysate was centrifuged for 30 min at 40.000 rpm at 4°C. The supernatant was incubated with Ni-NTA agarose (Qiagen, Hilden, Germany) for 2 h at 4°C on an end-over-end shaker. Agarose beads were transferred to micro-spin chromatography columns (Bio-Rad, Hercules, CA) followed by two washing steps with both, buffer I (50 mM NaH_2_PO_4_ (pH 8.0), 300 mM NaCl, 10 mM imidazole) and buffer II (50 mM NaH_2_PO_4_ (pH 8.0), 300 mM NaCl, 20 mM imidazole), followed by elution of the 6xHis-tagged GLEA2 protein with elution buffer (50 mM NaH_2_PO_4_ (pH 8.0), 300 mM NaCl, 250 mM imidazole).

### Enzyme-linked immunosorbent assay

For assessment of GLEA2 status, 6xHis-tagged GLEA2 protein (1 µg/ml in 0,2% BSA/PBS) was immobilized on Ni-NTA HisSorb Strips (Qiagen) overnight at 4°C. As a control we used 1 µg/ml BSA. Strips were four times washed with PBS containing 0,05% Tween 20 (PBST) for 30 sec. Serum samples were diluted 1?10 in 0,2% BSA/PBS and incubated overnight at 4°C followed by four washing steps. For detection horseradish peroxidase conjugated anti human IgG antibody (Dianova, Hamburg, Germany) was incubated 45 min at ambient temperature. Strips were washed 2× 30 s with PBST, 2× 30 s with PBS and dried on paper towels. Development was performed with 3,3,5,5-Tetramethyl-benzidine (TMB) (MP Biomedicals, Illkirch, France) substrate for 15–30 min. The reaction was stopped with 2 M H_2_SO_4_ and the absorption was monitored at 450/690 nm with an ELISA reader. ELISA assay was performed with 24 glioblastoma sera, 30 lung carcinoma sera and 30 sera from healthy donors.

### Statistical methods

We analyzed whether the changes of the GLEA2 levels between two consecutive measurement points in our time course are statistically significant. To this end, we applied unpaired two-tailed Wilcoxon Mann-Whitney test on our ELISA values. Alterations with a p-value <0.05 were considered as statistically significant.

## Results

### Threshold determination and separation of glioblastoma and control sera

Our previous study on the seroreactivity of GLEA2 was performed by SEREX or by spot assays. Since these approaches do not allow a quantitative analysis we studied the seroreactivity of GLEA2 by ELISA. In detail, we expressed N-terminal 6xHis-tagged GLEA2 in a baculovirus expression system to permit eukaryotic posttranslational modification of GLEA2. We analyzed the serostatus of 24 glioblastoma patients from the time prior to surgery throughout the further development of their disease. As control we included 30 healthy volunteers as well as 30 lung cancer patients. Recently, abundantly expression of GLEA2 was found in lung carcinoma of patients who underwent excessive chemotherapy [14]. As negative control for the ELISA assay, we used Ni-NTA strips treated with BSA only. We obtained readout values between 0 and 0.587. We empirically determined a threshold of 0.1 to achieve the best separation between glioblastoma patients and controls.

**Figure 1:**
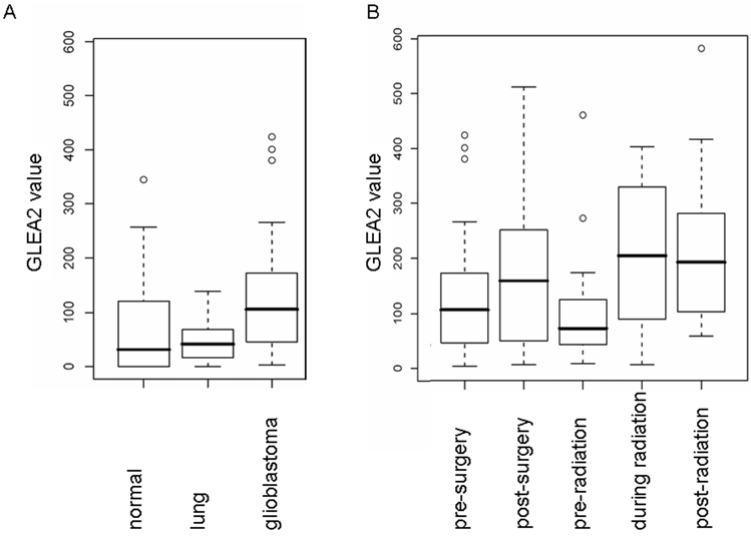
GLEA2 seroreactivity. (A) Comparison of GLEA2 seroreactivity between glioblastoma patients, healthy volunteers, and lung cancer patients as control groups. The differences between the controls and the glioblastoma patients group were statistically significant. The difference between the two control groups was not significant. (B) Comparison of GLEA2 seroreactivity between glioblastoma patients prior to surgery, after surgery, prior to radiation, during radiation and after radiation. The differences were significant between pre-radiation and during radiation. Black bars correspond to median values, the dazed lines to the 75% quantiles, circles represent outliers.

The difference of GLEA2 values between the controls and the glioblastoma patients was statistically significant as shown by unpaired two-tailed Wilcoxon Mann-Whitney test (glioblastoma vs. normal p-value 0.017; glioblastoma vs. lung p-value 0.0021). In contrast the difference between lung cancer patients and healthy volunteers was statistically not significant (p-value 0.92). The Box-Whisker-Plot in Figure 1A provides the median and interquantile range of the ELISA data for normal sera, lung cancer sera and the glioblastoma sera that were obtained before surgery.

In addition, we calculated the percentage of patients with GLEA2 ELISA values above the chosen threshold. In the following, this number is referred to as seroreactivity frequency. We obtained an overall seroreactivity frequency of 52.4% for glioblastoma patients. Previously we described an antibody response against GLEA2 in 48.4% of glioblastoma patients using SEREX and spot assays (13). The slight difference is likely due to different techniques used in both studies.

### GLEA2 seroreactivity course

To follow up on the course of the overall GLEA2 seroreactivity, we analyzed 24 glioblastoma patients not only prior to surgery, but also at different times after surgery. The second measurement was immediately after surgery, the third measurement prior to radiotherapy, the fourth measurement during radiotherapy and the fifth measurement after radiation.

The GLEA2 read out values of the five measurements are provided as Box-Whisker-Plot (Fig. 1B). As shown by this figure, the average seroreactivity increases shortly after surgery. We found the lowest overall frequency after surgery and prior to radiation. The highest average seroreactivity was found during radiotherapy and subsequent to radiotherapy. The only statistically significant difference between two consecutive measurement points was between prior to radiation and during radiation (p-value of 0.014).

For each time point we also calculated the percentage of patients with GLEA2 ELISA values above the chosen threshold. As stated above, we obtained an overall seroreactivity frequency of 52.4% for the time point prior to surgery. For the time point immediately after surgery we obtained an overall seroreactivity frequency of 65.2% and for the time point prior to radiation a frequency of 36.8%. During and after radiation we found more than 60% of patients with GLEA2 ELISA values above the chosen threshold.

### Increase of GLEA2 seroreactivity after radiotherapy

**Figure 2:**
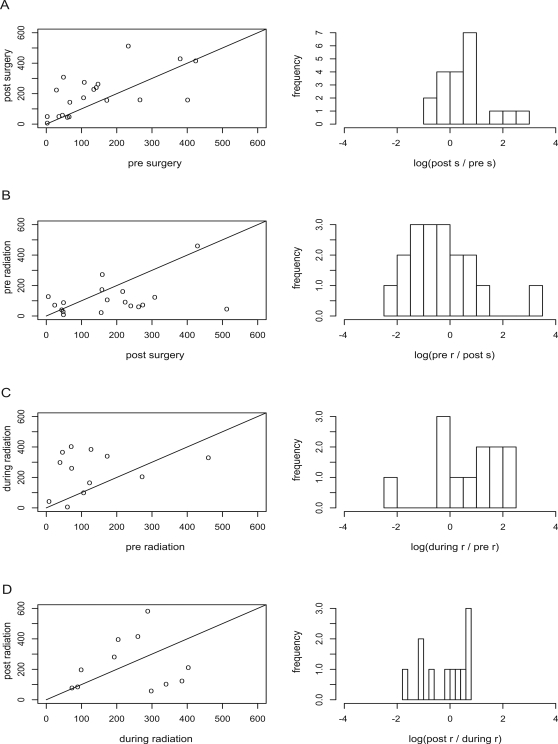
Individual course of GLEA2 seroreactivity. Each mark represents the GLEA2 value of a single patient. (A) Comparison of GLEA2 seroreactivities prior and after surgery. (B) Comparison of GLEA2 seroreactivities after surgery and prior to radiation. (C) Comparison of GLEA2 seroreactivities prior and during radiation. (D) Comparison of GLEA2 seroreactivities during and after radiation. On the right of each figure, a histogram of the quotient of GLEA2 values at the two time points is shown.

To follow up the GLEA2 seroreactivity of each individual patient, we compared GLEA2 values between two consecutive time points. For one third of the patients the GLEA2 values were similar before and after surgery. In Figure 2A, GLEA2 values on or near the diagonal indicate these cases. For the majority of the remaining cases we found an increase of GLEA2 values after surgery visualized by GLEA2 values above the diagonal in Figure 2A. For the minority of cases we found a decrease in GLEA2 values after surgery visualized by the values below the diagonal. Likewise, we compared the GLEA2 values after surgery and prior to radiation for each patient. As demonstrated in Figure 2B we found more GLEA2 values below the diagonal than above indicating a decrease of GLEA2 values. Comparing the GLEA2 values before and during radiotherapy, we found the majority of the values above the diagonal indicating a strong increase of GLEA2 seroreactivity (Figure 2C). Comparing the GLEA2 values during and after radiation we found similar numbers of GLEA2 values above or below the diagonal indicating that GLEA2 values did not obviously change after radiation (Fig. 2D). To compute significance values for consecutive points in time, we performed an unpaired two tailed Wilcoxon Mann-Whitney tests. Consistent with the analysis of the overall GLEA2 seroreactivity frequency, the differences between GLEA2 values prior to radiation and during radiation were significant (p-value of 0.014).

### Individual GLEA2 course

We attempted to group the GLEA2 ELISA values according to their time course. We found five patients that had ELISA values above the threshold throughout the time, and four patients that had values below the threshold throughout the time. The remaining 15 patients showed GLEA2 values that changed over time. Out of seven patients that had high GLEA2 values prior to surgery, five patients showed decreased GLEA2 values prior to radiation. Out of these five patients, three showed again increased GLEA2 values during and after radiation. Out of seven patients that had low GLEA2 values prior to surgery, six had also increased GLEA2 values during and after radiation.

### GLEA2 course of seropositive and seronegative patients

**Figure 3:**
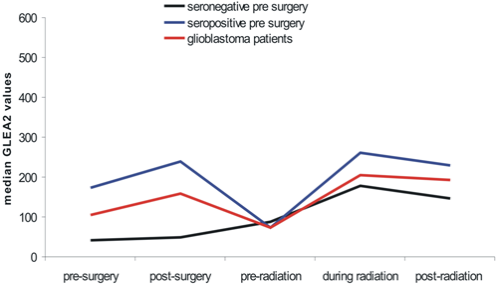
Main course of GLEA2 seroreactivity. Line plot of median GLEA2 values on the five measurement times. The median values of all 24 glioblastoma patients (red line) were compared to two patient subgroups. Patients that were GLEA2 positive prior to surgery were indicated by blue line. Patients that were GLEA2 negative prior to surgery were indicated by black line.

We compared the median GLEA2 values between patients that were negative for GLEA2 autoantibodies prior to surgery and patients that were positive for GLEA2 autoantibodies prior to surgery. GLEA2 postive patients showed a strong decline of GLEA2 values after surgery followed by an increase of GLEA2 values to a higher level during radiation. After surgery, GLEA2 negative patients showed GLEA2 levels similar to the levels of GLEA2 positive patients. During radiation, GLEA2 negative patients showed an increase of GLEA2 values. This increase was however lower than the increase found for GLEA2 positive patients. Both, GLEA2 positive and GLEA2 negative patients showed a slight decline of the GLEA2 values after radiation (Figure 3). We also compared the median GLEA2 values between patients that were treated by different regimens of temozolomide and patients that were treated by radiotherapy only. We did not found any statistically significant difference of GLEA2 values between these patients groups.

## Discussion

Recent evidence indicated modulation of the MHC class I expression and the peptide repertoire by ionizing radiation. Radiation of a melanoma cell line lead to increased peptide production and antigen presentation [5]. Immunotherapy allowed eradication of a murine colon adenocarcinoma only when preceded by radiotherapy of the tumor tissue [5]. These results lead us to ask how radiation influences the seroreactivity of antigens in human tumor patients. We chose to analyze glioblastoma patients that have a very poor prognosis despite surgery and radiotherapy. In addition, immunotherapy for tumors located in the CNS has generally not achieved the results reported for some peripherally located tumors. The relative paucity of glioma-associated antigens that can be targeted by the immune system may partially account for this situation [8]–[13].

Our results provide first *in vivo* evidence that radiation may increase the frequency of the autologous antibody response against human tumor antigens. Without further experimental evidence, we can, however, not rule out that the increased GLEA2 values are due to other factors but radiation. We were not able to compare the GLEA2 values between patients with and without radiation. The majority of glioblastoma patients undergoes radiotherapy after surgery even in clinical trials like the phase III NOA-8 trial and patients who are not treated by radiation generally showed a Karnofsky Performance Score lower 60.

However several observations lead us to conclude that the increase in GLEA2 autoantibodies is likely due to radiation. In general we found an increase of GLEA2 values during radiation and a subsequent decrease of GLEA2 values after radiation. It appears unlikely that these GLEA2 values are due to tumor growth pattern: The median residual tumor volume after gross-total removal is reduced to 12% as shown by quantitative radiological analysis [15]. Provided the mean tumor doubling time of glioblastoma is 24 days [16], the GLEA2 measurements prior to radiation were performed after 0.13 doubling times. The measurements during and after radiation were performed after 2.0 and 5.5 doubling times. The resulting increase of tumor volume does not readily explain the strong increase of GLEA2 autoantibodies during radiation and the slight decrease after radiation.

It is also conceivable that radiation can elicit an antibody response against novel antigens that are not immunogenic in glioma patients without radiation. Our finding is in keeping with this idea since GLEA2 was immunogenic in some patients only during or after but not prior to radiation. In the future, radiation may significantly improve the results of immunotherapy for tumors located in the CNS. Modulating the tumor immunoresponse can contribute to overcome the current shortcomings of immunotherapy.

Our results provide also evidence that the analysis of antigen seroreactivity may be useful for monitoring tumor development under treatment. The immune system may be ideally suited to identify even subtle changes in tumor development that cannot be picked up by other approaches. Future developments can combine the power of imaging technology and the sensitivity of approaches that utilize the immune system for tumor detection. Detection and monitoring of human tumors by seroreactivity of antigens is, however, still in its infancy. Out of the over 2000 antigens known to be immunogenic in human tumor patients, only very few have been analyzed for the course of their seroreactivity during tumor progression under treatment [17]. There are no studies that follow the antigen reactivity during the progress of human brain tumors. Surpassing the scope of our study, an optimized monitoring of brain tumor development under treatment would require an extended number of immunogenic antigens that yet need to be identified.

Our study does not focus on the question why radiation results in increased GLEA2 seroreactivity. One obvious explanation is the release of GLEA2 as the result of cell destruction due to radiation. The increased GLEA2 seroreactivity found in glioblastoma patients at the time of surgery may also be caused by necrosis that is typically associated with human glioblastoma. Alternatively, GLEA2 may also be presented via MHC class I on the surface of glioblastoma cells [5]. However, MHC class I antigen presentation is remarkably inefficient. This may be overcome by radiation that both enhance degradation of cellular proteins and MHC class I expression.

Independent of the molecular mechanisms, the study indicates that radiation increases the antibody response against GLEA2 in glioma patients. Although obtaining blood at defined time points after surgery may be difficult for a larger number of glioblastoma patients, our study provides strong evidence that it is worthwhile (a) to identify further antigens that are immunogenic in glioblastoma patients and (b) to follow the course of their seroreactivity during glioma development under treatment. Only the analysis of seroreactivity over longer periods can fully reveal the value of immunogenic antigens for tumor monitoring.
